# Evolution of a Disease Surveillance System: An Increase in Reporting of Human Monkeypox Disease in the Democratic Republic of the Congo, 2001–2013

**DOI:** 10.9734/IJTDH/2017/35885

**Published:** 2017-08-28

**Authors:** Nicole A. Hoff, Reena H. Doshi, Brian Colwell, Benoit Kebela-Illunga, Patrick Mukadi, Mathias Mossoko, D’Andre Spencer, Jean-Jacque Muyembe-Tamfum, Emile Okitolonda-Wemakoy, Jamie Lloyd-Smith, Anne W. Rimoin

**Affiliations:** 1 Fielding School of Public Health, University of California, Los Angeles, CA, USA; 2 Health Promotion and Community Health Sciences, Texas A&M School of Public Health, College Station, TX, USA; 3 Ministry of Public Health, Direction for Disease Control, Democratic Republic of the Congo; 4 National Institute for Biomedical Research, Democratic Republic of the Congo; 5 Kinshasa School of Public Health, University of Kinshasa, Democratic Republic of the Congo; 6 University of California, Los Angeles, CA, USA

**Keywords:** Monkeypox, passive surveillance, disease trends, Democratic Republic of Congo

## Abstract

**Objective:**

Evaluating the effectiveness of a surveillance system, and how it improves over time has significant implications for disease control and prevention. In the Democratic Republic of Congo (DRC), the Integrated Disease Surveillance and Response (IDSR) was implemented to estimate the burden of disease, monitor changes in disease occurrence, and inform resource allocation. For this effort we utilized national passive surveillance data from DRC’s IDSR to explore reporting trends of human monkeypox (MPX) from 2001 to 2013.

**Methods:**

We obtained surveillance data on MPX cases occurring between January 2001 and December 2013 from the DRC Ministry of Health (MoH). Phases of the surveillance system, yearly trends in reporting and estimated incidence for MPX were analyzed using SAS v9.2 and Health Mapper.

**Results:**

Between 2001 and 2013, three discrete surveillance phases were identified that described the evolution of the surveillance system. Overall, an increase in suspected MPX cases was reported, beyond what would be expected from simply an improved reporting system. When restricting the analysis to the “stable phase,” national estimated incidence increased from 2.13 per 100,000 in 2008 to 2.84 per 100,000 in 2013.

**Conclusions:**

The reported increase in MPX, based on an evolving surveillance system, is likely to be a true increase in disease occurrence rather than simply improvements to the surveillance system. Further analyses should provide critical information for improved prevention and control strategies and highlight areas of improvement for future data collection efforts.

## 1. INTRODUCTION

The systematic collection of surveillance data has significant implications for effective disease control and prevention [1]. Well-functioning surveillance systems can be used to estimate the burden of disease, monitor changes in disease occurrence, assess geographic spread, identify high-risk populations and other health concerns, and inform resource allocation [2,3]. Therefore, evaluating the effectiveness of surveillance systems, and how they improve over time, is a critical health imperative.

In 1998, the World Health Organization’s Regional Office for Africa (WHO-AFRO) established the Integrated Disease Surveillance and Response (IDSR) unit to strengthen public health surveillance and disease response in a number of African countries [3–5]. Diseases with epidemic potential or those targeted for elimination/eradication are considered notifiable in the IDSR unit, and each individual country may incorporate other diseases of public health importance that require reporting [6]. This surveillance system relies on passive collection of data sent from health care facilities throughout each country on a weekly basis [7].

In the Democratic Republic of Congo (DRC), the IDSR was implemented in 2000 under the Ministry of Health (MoH) Direction for Disease Control (DLM). Implementation of an effective surveillance system, however, has been challenging. Decades of political and social instability have resulted in the deterioration of the health care system [8,9]. The country continues to recover from a multi-year civil conflict that left many areas without modern roads or transportation and produced more than one million refugees and internally displaced persons [10]. Moreover, cross-border incursions and rebel insurgencies continue to occur in the eastern part of the country. These obstacles make communication and supervision of local health centers, as well as disease surveillance and reporting, extremely difficult. Much of the country’s inaccessible terrain is heavily forested and has been identified as ideal geographic locations for emergence of viral diseases, including human monkeypox (MPX) [11].

Monkeypox is a zoonotic viral infection found in a variety of mammals, including humans [11,12]. It is indigenous to the Congo River Basin and is endemic among a variety of wild animals including rodents and monkeys, which are the primary vectors to humans [13,14]. When humans are infected with MPX they typically develop a less severe smallpox-like illness that includes a fever and pustules, which usually presents in extremities (feet, hands, and face) and crust over after 10 days [12,15]. A large majority of worldwide cases are reported in DRC, where the disease is endemic in forest animals with frequent spillover into the human population [11]. While MPX has been an IDSR reportable disease in DRC since 2001, the true burden remains largely unknown, with no reliable national estimates, likely because MPX cases often occur in remote locations that are difficult to access. Underreporting is common, and diagnosis in the field cannot be confirmed without polymerase chain reaction (PCR) testing, presenting challenges for conducting research based on the ecology, epidemiology, natural history, and pathogenesis of the infection [16–18].

Despite the challenges, surveillance data can be used to assess disease incidence trends over time to inform policy in resource limited settings [19]. Therefore, we utilized national passive surveillance data from DRC’s IDSR to explore reporting trends of MPX from 2001 to 2013. This paper examines one system in the Democratic Republic of the Congo (DRC) and how monkeypox surveillance has developed over the last 14 years.

## 2. METHODS

### 2.1 Suspected MPX Case Counts

MPX is one of 13 reportable diseases in DRC’s IDSR [20]. The definition of a suspected case of MPX has remained unchanged since its inclusion in the IDSR in 2001: “any person appearing with a sudden onset of high fever, followed a few days later by a vesicular-pustule eruption presenting predominantly on the face, palms of the hands, and soles of the feet; or the presence of at least 5 smallpox type scabs” [20].

Suspected MPX cases and deaths are reported weekly to each of the 516 health zones in 11 provinces [20]. Since these data were collected the nation has been divided into 26 separate provinces, but this analysis will refer to the old 11 provinces. Across the country, over 10,000 health centers are required to send weekly written reports of suspected MPX case counts [20]. Reported information also includes province, district (composed of about 5–10 health zones), health zone and patient age group (0–11 months, 12–59 months, and 5 years +).

### 2.2 Descriptive Analyses

MPX case counts, estimated incidence rates, and 95% confidence intervals were calculated at the national and provincial levels. A negative binomial distribution was used to account for over-dispersion with the logarithm of the yearly population as an offset variable. The proportion of health zones reporting MPX was calculated by dividing the number of health zones reporting ≥ 1 MPX case during a given year by the number of heath zones reporting any reportable disease in the same year (Table 1). Incidence was defined as number of cases reported by health zone over the estimated population for that zone. We utilized Expanded Programme on Immunization (EPI) population estimates, which provided the only available health zone level population data [18,21–23]. Duplicate entries with the same health zone name and epidemiological week were removed. All analyses were performed using SAS v9.4 [24] and maps were created using Health Mapper 4.3 [25].

### 2.3 Changes in Disease Reporting

We defined three conceptual phases of the passive reporting system based on historical events and changes to the surveillance system: 1) Implementation Phase (2001–2003): the program was not fully implemented due to widespread political instability, and few health zones were reporting; 2) Adjustment Phase (2004–2007): political instability was reduced and there was increased and consistent reporting of diseases throughout the country; and 3) Stable Phase (2008–2013): almost all health zones were reporting regularly, and there were no major changes to the reporting system. We conducted t-tests of mean incidence by implementation phase.

In order to estimate the changes in reporting over time, we utilized suspected Acute Flaccid Paralysis (AFP) and tetanus case counts reported to the IDSR for pattern comparison, because both of these diseases were expected to have constant background reporting rates. AFP was selected based on a consistent background rate (2 cases per 100,000 population), [26] while reporting of tetanus was expected to remain stable or decrease with increasing immunization [27]. Mean annual percent change in disease incidence was calculated using generalized linear models (GLM). Sensitivity analyses were performed by removing the Tshuapa (12 health zones) and Sankuru (12 health zones) districts from the available data, as active MPX surveillance had been implemented in both districts to determine if areas with active surveillance was the main factor for increase in disease reporting.

## 3. RESULTS

### 3.1 Overall MPX Trends

**Fig. 1:**
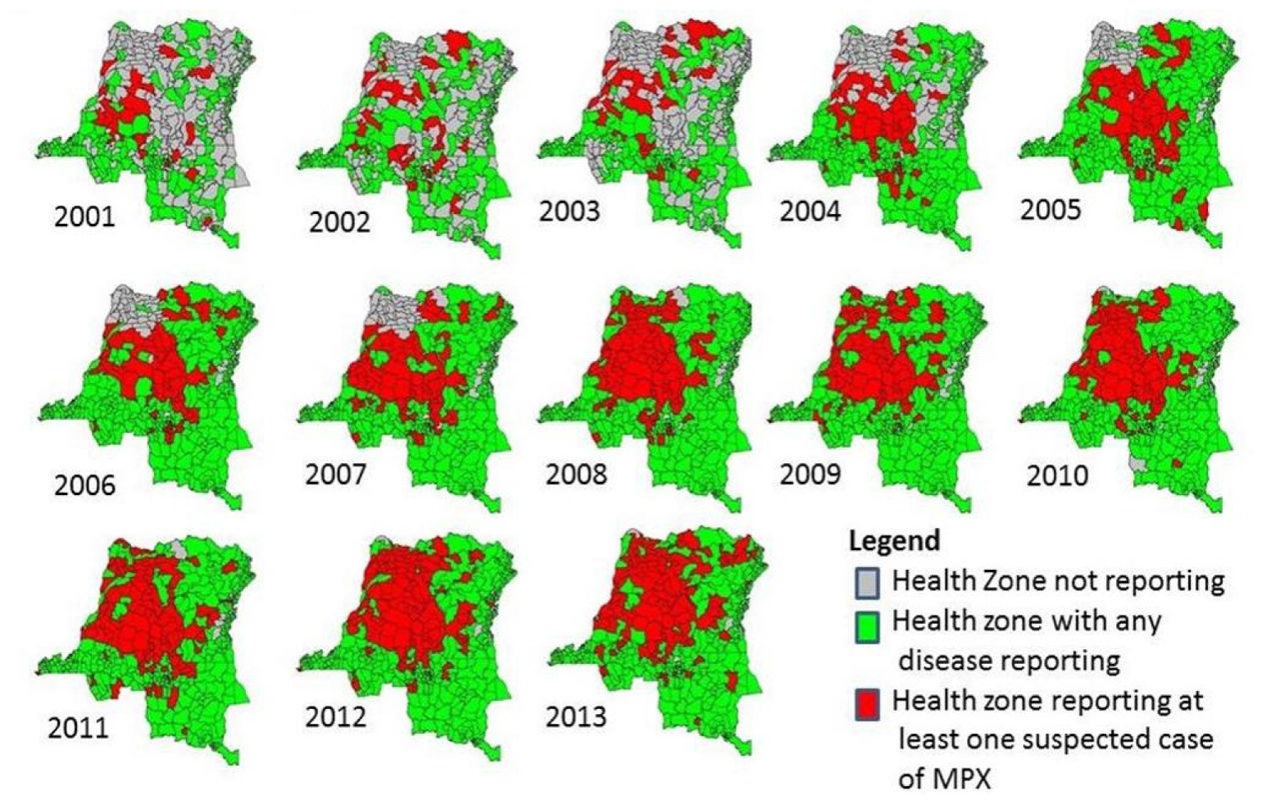
Disease reporting to the IDSR, 2001–2013

From 2001 to 2013 the number of health zones reporting any reportable disease increased from 253 to 514. During the same time period 19,646 suspected MPX cases reported and the number of health zones reporting a case increased from 31 to 136 (Table 1). The lowest reported incidence for suspected MPX cases was in 2001(0.64 per 100,000) and the highest was in 2012 (3.11 per 100,000 persons). This observation remained true after removal of the two active surveillance areas (0.61 and 2.0 per 100,000 persons, respectively) (Table 2). Suspected cases of MPX were most commonly reported in the northern and central portion of the country (Fig. 1). Equateur province had the highest mean incidence over the 13 years, as well as highest annual reported incidence, of suspected MPX cases, followed by Kasai Oriental and Maniema provinces (Table 3). From 2001 to 2013 there was a significant increase in reported cases of MPX (p<0.001). This trend remained significant after the removal of the Tshuapa and Sankuru districts (Table 4).

### 3.2 Phases of the IDSR

#### 3.2.1 Implementation phase

**Fig. 2:**
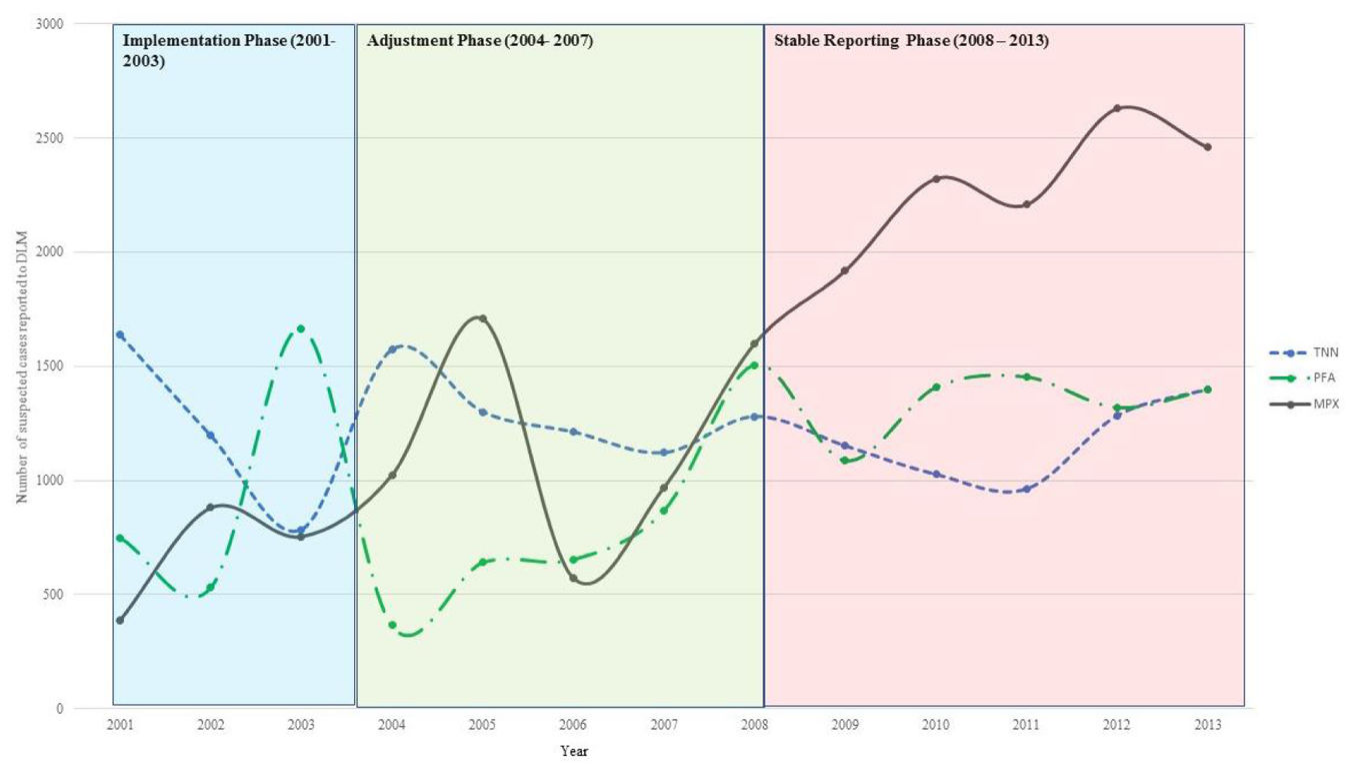
Disease reporting to the IDSR, 2001–2014

We considered the years 2001–2003 the “implementation phase.” The IDSR had recently been introduced and MPX, AFP, and tetanus were all included as reportable diseases. During this phase, a total of 2,024 suspected MPX cases were reported. Kasai Occidental and Bandundu Provinces had the highest incidence (2.70 per 100,000 persons and 2.20 per 100,000 persons, respectively). During this phase, there was high variation in AFP and tetanus incidence (Fig. 2), leading us to conclude that there would be similar instability in the reporting for MPX.

#### 3.2.2 Adjustment phase

We considered 2004 to 2007 the “adjustment phase.” The implementation and adjustment phase combined incidence based on a chi-square test were not significantly different (p=0.54, data not shown). If a health zone reported case counts for at least one disease during an epidemiologic week, we assumed all other diseases with missing case counts to have zero cases. During this phase, the IDSR integrated additional variables, including age categories, case-fatality rates, and health zone populations. This time also marked the end of widespread civil unrest.

Between 2005 and 2006, there was a sharp increase, followed by a decrease in the national estimated MPX incidence (2.48 per 100,000 persons to 1.11 per 100,000 persons) (Table 2). Both Kasai Oriental and Equateur provinces had similar trends. In comparison, tetanus reporting remained relatively stable while there was continual fluctuation of AFP reporting, however these changes were not significant (2001–2007: p=0.586 (tetanus) and p=0.182 (AFP)) (Table 4).

#### 3.2.3 Stable phase

We consider 2008–2013, the “stable reporting phase.” By 2008, there were 515 health zones (a 516^th^ was added in 2012), with 502 of those reporting at least one case of any reportable disease during the year compared to 464 the year before. Both the implementation and adjustment phase differed significantly in the mean number of suspected cases reported yearly from the stable phase (p<0.05) (Table 4). We again assumed all other diseases with missing case counts to have zero cases for other reportable diseases when health zones reported cases for at least one disease each week. The number of health zones reporting any disease stayed fairly stable, and there were no changes to the case definitions of the 15 reportable diseases. A second active surveillance program was implemented in 2008, in the Tshuapa District (comprised of 12 health zones) of the Equateur province. As Table 3 indicates, this led to increased incidence of suspected MPX reported in the district.

Reported MPX incidence between 2008 and 2013 (2.13 to 2.84 per 100,000, respectively) increased significantly, with an estimated change in incidence per year of 6.2% (95% C.I.: 2.0%, 9.4%, p=0.002). Equateur and Kasai Oriental provinces had the highest incidence for 2013 (12.83 and 5.78 per 100,000, persons respectively). The predicted trend in incidence remained significant after removal of the Sankuru and Tshuapa Districts (p<0.001), which had superior health care worker training on recognition of MPX disease and reporting requirements. While the number of health zones reporting any disease during this time period increased by 12 (502 to 514), there were 5 additional health zones reporting at least 1 case of suspected MPX, indicating that it may be spreading geographically (Table 1). Simultaneously, the estimated change in annual incidence for AFP indicated a slight decrease, but it was not significant (−3.0%, 95% C.I.: −6.8%, 2.0%, p=0.297). The same trend was seen for tetanus, which showed a slight but non-significant decrease from 2008 to 2013 (−1.0%, 95% C.I.: −6.8%, 5.1%, p=0.756) (Table 4).

## 4. DISCUSSION

The results suggest that reported MPX incidence increased between 2001 and 2013 and that the increase cannot be explained solely by improvements to the surveillance system. While an active MPX surveillance program conducted from 1981–1986 by WHO suggested that the observed increase in MPX incidence was a result of strengthened surveillance activities, our evaluation 15 years later suggests a real increase [12]. Between 2001 and 2013, there was an almost 4-fold increase in estimated MPX incidence, with the largest increase during the stable phase. Moreover, the consistency of AFP and tetanus reporting in the same health zones and years provide more evidence for this increase.

Our analyses highlighted three distinct phases since IDSR implementation in 2001. During the “implementation” phase, there were inconsistencies in disease reporting and fewer health zones reporting cases (289 in 2003 to 514 in 2013). During the adjustment phase, the number of health zones reporting diseases increased substantially, however, reporting gaps remained, notably in the northwestern region of the country. Confusion with the collection of additional variables and the declaration of the end of the second war may partially explain inconsistencies. While intertribal and rebel group fighting continued to occur, the declaration increased stability within the country [10]. By 2008, almost all health zones were regularly reporting to the IDSR, and there were no additional changes to the system. During the stable phase (2008–2013), we saw the largest increase in MPX reporting.

A number of factors may be contributing to the observed increase in MPX incidence: (1) increasing vaccinia-naïve populations; (2) decreasing immunity among previously vaccinated persons; [18] and (3) increased dependence on bush meat as a regular source of nutritional sustenance [28–30]. While misclassification of other rash illnesses reported to the IDSR could lead to an artificial increase in estimated MPX incidence, MPX specimens tested at the National Institute for Biomedical Research (INRB) should be used to further validate our results.

During the stable phase, 17 additional health zones reported at least one suspected case of MPX – a 2.8% increase from 2008 to 2013. This could be indicative of not only an increase in MPX incidence, but also an expanding geographic distribution of disease. Ecological niche modeling supports this hypothesis and suggests that the distribution of MPX cases could extend to most of the country, including the eastern provinces in less heavily forested areas [17,16,31].

Our results are consistent with an active surveillance program conducted between 2005 and 2007 in the Sankuru District, which suggested an increase in incidence compared to the 1980’s surveillance [18]. Our estimated incidence was lower than those found in the Sankuru District, likely explained by the active case-finding methodology employed.

Between 2005 and 2006, there was an unexpectedly sharp increase, then decrease in cases reported to the IDSR. However, based on the active surveillance system in the Sankuru District, there was an increase in 2006 in the number of samples collected and confirmed at the national laboratory [18]. This suggests that surveillance may have been focused on specimen collection rather than passive reporting in 2006; however by 2007 more cases were reported to the IDSR system.

Our analyses are subject to a number of limitations. Case reporting is likely to be severely underestimated, data completeness is questionable and little information is collected beyond age data. While over 10,000 health centers are required to report weekly case counts to their respective health zone offices, only a small proportion of them consistently send the data. Additionally, traditional healers, prayer houses, and privately-run clinics are not always required to participate in case reporting [32]. While, the INRB collects samples for laboratory analysis from health zones with suspected MPX cases, linkage to the IDSR system remains incomplete.

There is likely to be substantial disease misclassification, as the case-definition is non-specific, and thus may have biased our results. For example, Varicella meets the MPX case definition, and several rash illnesses are sometimes misdiagnosed as MPX, possibly inflating the estimated incidence. There is no reason, however, to believe that misclassification is occurring at a higher rate in recent years than when the reporting system was initiated. An unavoidable weakness is that incidence estimates are likely to be biased due to inaccurate population estimates [18,21–23]. Large-scale population movement during the past two decades due to civil conflict and transient rural populations in the forest or near the rivers could impact population estimates, which are currently based on the most recent available data – 1984 census. We attempted to reduce the likelihood of this through use of the standard estimate of population growth in the DRC [21].

The use of aggregated data limits the ability to make causal inferences on an individual’s risk of disease [33]. Ecologic bias, disease and exposure misclassification within groups, temporal ambiguity, and an inability to control for all confounders can lead to significant bias [33]. We did not have data on all potential confounders, including age, sex, positive confirmation of cases, and if the cases were due to animal or human contact, which could affect our estimated MPX incidence [33].

Given the limited resources in DRC, extensive analyses utilizing the surveillance data are rarely accomplished. Despite these structural and reporting limitations, we still observed a significant increase in estimated MPX incidence and an expansion of the geographic distribution of disease. Based on our analyses, additional research should target provinces with the highest estimated incidence: Equateur, Kasai Oriental, Orientale, and Maniema. While small-scale programs targeting clinical characteristics and individual risk factors to MPX have already been implemented in specific districts, [18,34] more research is needed to determine the major ecologic and behavioral factors contributing to the observed increase in estimated incidence, which could include a better understanding of the changing bush meat trading system. For areas lacking targeted interventions, but where estimated MPX incidence has increased, evaluations should be undertaken to determine if an active surveillance system is necessary or if sentinel surveillance sites should be established. Further, trends identified though our analysis could be used to develop triggers which could alert the MOH to increased occurrence over a period of time.

## 5. CONCLUSION

In the DRC, there is a need for additional investment at the operational level to strengthen passive reporting. While the IDSR reached a stable phase in 2008, many health zones are still limited in their ability to submit weekly reports in a timely manner, thus leading to possible delays in outbreak notification and containment. More streamlined reporting methodology, increased feedback from the national to the local level and improved linkage between passive and the case-based surveillance systems will be necessary as we aim improve our understanding of MPX disease occurrence and distribution. Improved surveillance systems will be essential in the containment of future outbreaks as these systems will be necessary in the detection and reaction to potential outbreaks. Effective surveillance systems will in part provide early warning alerts and needed information for healthy systems to adequate targeted interventions. These improvements could lead to a broader impact on the surveillance system as a whole for the reporting of other emerging pathogens and diseases targeted for eradication in the DRC and Central Africa

## Figures and Tables

**Table 1:** Number of health zones reporting suspected monkeypox cases to the IDSR, 2001–2013

Year	# Suspected cases	# HZ Reporting 1 or more MPX	# HZ reporting any disease	% Reporting MPX^1^
2001	388	31	253	12.3
2002	881	50	292	17.1
2003	755	44	295	14.9
2004	1024	77	374	20.6
2005	1708	83	454	18.3
2006	783	76	464	16.4
2007	970	90	464	19.4
2008	1599	119	502	23.7
2009	1919	108	502	21.5
2010	2322	107	504	21.2
2011	2208	123	507	24.3
2012	2629	133	508	26.2
2013	2460	136	514	26.5
Total	19646	264	514	52.5

1 % Reporting MPX was calculated by the number of health zones reporting more than one case of MPX during a given year divided by the number of heath zones reported any reportable disease in a given year

**Table 2:** Suspected MPX incidence in DRC (with and without the active surveillance areas), 2001–2013

Year	Incidence, per 100,000, 95% CI	Incidence per 100,000 (without active surveillance areas), 95% CI
2001	0.64 (0.09, 4.50)	0.61 (0.09, 4.30)
2002	1.4 (0.20, 9.90)	0.94 (0.13, 6.70)
2003	1.16 (0.16, 8.30)	0.72 (0.10, 5.10)
2004	1.53 (0.22, 10.90)	0.82 (0.12, 5.80)
2005	2.48 (0.35, 17.60)	0.96 (0.13, 6.80)
2006	1.11 (0.16, 7.80)	0.67 (0.09, 4.80)
2007	1.33 (0.19, 9.40)	0.60 (0.08, 4.20)
2008	2.13 (0.30, 15.10)	1.00 (0.15, 7.40)
2009	2.48 (0.35, 17.60)	1.20 (0.17, 8.80)
2010	2.91 (0.42, 20.70)	1.40 (0.19, 9.60)
2011	2.69 (0.38, 19.10)	1.60 (0.23, 11.60)
2012	3.11 (0.44, 22.10)	2.00 (0.28, 14.40)
2013	2.82 (0.40, 20.10)	1.50 (0.22, 10.90)

**Table 3:** Suspected MPX incidence in selected provinces of DRC, 2001–2013

Year	Equateur^1^,^2^	Kasai Oriental^1^,^3^	Maniema^1^	Bandundu^1^	Oriental^1^	Kasai Occidental^1^
2001	0.53	0.83	0.06	2.20	0.07	2.70
2002	6.58	3.84	0.06	0.88	0.26	0.49
2003	4.60	0.16	0.48	1.66	3.23	0.34
2004	3.40	4.94	0.17	1.59	1.74	1.08
2005	8.29	11.26	1.01	0.20	0.82	0.52
2006	2.48	3.66	0.82	1.23	2.07	0.06
2007	2.03	6.20	0.48	1.49	0.83	1.45
2008	9.66	4.82	0.41	1.71	0.79	1.86
2009	10.71	5.96	3.55	3.32	0.54	0.65
2010	12.19	7.91	7.39	3.22	0.34	0.36
2011	10.98	4.43	6.98	3.14	2.64	0.75
2012	11.02	6.88	5.96	2.78	2.19	3.67
2013	12.83	5.78	2.67	1.52	1.90	2.25
Total	7.69	5.25	2.53	1.91	1.37	1.28

1 Incidence: per 100,000 persons

2 Tshuapa District located in this Province

3 Sankuru District located in this Province

**Table 4:** Mean percent change in predicted yearly incidence for MPX, Tetanus, AFP (2001–2013, 2001–2007 and 2008–2013)

Disease reported	All years (2001–2013)			Phase 1+2 (Years 2001–2007)			Phase 3 (Years 2008–2013)		

	% Change	95% CI	P-value	% Change	95% CI	P-value	% Change	95% CI	P-value
**MPX (whole country)**	10.5	(6.2, 15.0)	<0.001	9.4	(−4.9, 25.9)	0.197	6.2	(2.0, 9.4)	0.002
**MPX (without active surveillance)**	8.3	(5.1, 11.6)	<0.001	−2.0	(−8.6, 5.1)	0.628	10.5	(5.1, 17.3)	<0.001
**AFP**	3.0	(−2.0, 8.3)	0.236	−4.9	(−18.9, 12.7)	0.586	−3.0	(−6.8, 2.0)	0.297
**Tetanus**	−3.9	(−6.8, −1.0)	0.003	−4.9	(−10.4, 2.0)	0.182	−1.0	(−6.8, 5.1)	0.756
